# RECRUITMENT OF FAMILY CAREGIVERS OF AFRICAN AMERICAN OLDER ADULTS WITH DEMENTIA

**DOI:** 10.1093/geroni/igz038.2197

**Published:** 2019-11-08

**Authors:** Karen O Moss, Kathy Wright, Glenna Brewster, Karen Rose, Celia E Wills, Todd Monroe

**Affiliations:** 1 The Ohio State University, Columbus, United States; 2 The Ohio State University, Columbus, Ohio, United States; 3 Emory University, Atlanta, Georgia, United States; 4 University of Tennessee, Knoxville, Knoxville, Tennessee, United States

## Abstract

Multiple factors influence decision-making of family caregivers of African American older adults with dementia about participation in end-of-life care research. Existing literature on best practices for research recruitment includes little on recruitment strategies for this specific population. The purpose of this presentation is to analyze successful recruitment strategies used with family caregivers of African American older adults with dementia. Sixty-five caregivers of African American older adults with dementia were recruited over 11 months throughout a southeastern state from local communities and Program of All-Inclusive Care for the Elderly. Community partnership strategies such as community-based networking, purposively-targeted presentations, and leveraging of existing social networks provided a strong basis for successful recruitment. These strategies were credible to the participant population and effective in engaging both research participants and community partner stakeholders in working on shared goals of improving older adult and caregiver outcomes near the end of life.

